# Update in the Diagnosis and Treatment of Root Canal Therapy in Temporary Dentition through Different Rotatory Systems: A Systematic Review

**DOI:** 10.3390/diagnostics12112775

**Published:** 2022-11-13

**Authors:** Mª Dolores Casaña Ruiz, Laura Marqués Martínez, Esther García Miralles

**Affiliations:** Dentistry Department, Faculty of Medicine and Health Sciences, Catholic University of Valencia San Vicente Mártir, 46001 Valencia, Spain

**Keywords:** primary teeth, pulpectomy, rotatory system, root canal treatment

## Abstract

The need to perform fast, effective and efficient pulpectomies has led to the development of numerous valid rotary systems. Its technical features allow the clinician to obtain good results in less working time. The objective of this study is to compare the characteristics of the different current rotary systems to favor a correct diagnosis and subsequent treatment. A systematic review of the literature has been carried out in accordance with the PRISMA recommendations. A search was carried out in PubMed, Embase Scopus, Cochrane and Web of Science databases, and was completed with a manual search. The following variables were extracted from the selected studies: author, year, sample, rotary systems used (length, diameter, taper, speed), obturation material and irrigant. From the initial electronic search of the five databases, 315 articles were identified. Once the duplicate articles were eliminated, a total of 233 remained. After reading both title and abstract, 200 articles were eliminated, leaving 33. On account of reading the full text, 22 were eliminated for not answering the research question or the inclusion criteria, leaving a total of 11 articles for the systematic review. Rotary systems which are able to adapt to the root anatomy of primary teeth and allow rapid and simple instrumentation, without producing excessive extrusion of debris at the root apex, will be the ones that provide the best results to the pediatric dentist during the performance of pulp treatment in primary teeth. Clinical success will only be achieved through proper prior diagnosis.

## 1. Introduction

Extensive caries at early ages can affect the pulp tissue causing pain and discomfort in children. Pulpectomy has greatly contributed to the preservation of necrotic primary teeth avoiding harmful habits and loss of space due to the migration of neighboring teeth [1].

Accurate diagnosis of the pulp status will be essential to select the most appropriate treatment in each case and thus guide the treatment plan. Therefore, a detailed pain history and meticulous clinical examination supplemented with a high-quality periapical radiograph and pulp sensibility testing using low-temperature cold testing are necessary to assess the status of the pulp [2].

Radiographic diagnosis should be used to assist clinical decision-making since it allows the assessment of the severity of the caries and the commitment of the supporting tissues. Depending on the severity of the caries, the appropriate projection between bitewing and periapical will have to be decided. For pulp treatments, periapical radiographs are the most recommended, since they allow assessing the affectation of the tooth in its entirety, the state of the bone, the distance to the pulp, the state of the roots and the distance to the germ of the permanent tooth. The routine use of CBCT is not justified for assessing pulpitis. The final indication for determining pulpal inflammation will be hemostasis, or lack thereof, that occurs during pulpal treatment [3]

The objectives for the treatment of root canals in temporary teeth lie in the disinfection of the root canals providing both an optimal seal to avoid reinfection and the allowance to restore functionality [4].

In pediatric endodontics, the unpredictability and difficulty of root canal anatomy of temporary teeth such as curved or claw roots, the length and irregularity of the canals, thinner dentin walls and above all, physiological resorption, adds to a clinician’s challenge [5].

In addition, success of the pulp treatment procedure mainly depends upon the speed of disinfection, the provision of optimal asepsis without weakening the walls or affecting the successor tooth germ and the guarantee that the tooth is conserved until its physiological exfoliation [6].

Classically, root canal preparation in pediatric patients has been performed with stainless steel manual files. However, there are certain limitations such as the possibility of generating alterations in the original shape of the root canals, as well as perforations due to their low flexibility. Furthermore, they may generate greater postoperative pain and unquestionably, a work time hazard [7].

In the 1980s, Ni-Ti rotary files were created with the aim of overcoming previous limitations, offering advantages such as a better conical shape and higher quality debridement, obturation in a short time due to their high flexibility, greater resistance to static cyclic fatigue, greater angle of torsion in the fracture and less torsion in general. Although slight differences are found depending on the system used, for example, One RECI (OR; Micromega, Besançon, France) or WaveOne Gold (WOG; Dentsply Maillefer, Ballaigues, Switzerland), all of them show mechanical characteristics of great utility in clinical practice [8].

Over the years, rotary Ni-Ti systems have been introduced with a wide range of designs and usage techniques, allowing for faster preparation while preserving the original shape, even of curved root canals [6].

The objective of this study is to update the different current rotary systems and the results obtained from their use for root canal treatment in temporary dentition in order to achieve an effective diagnosis and treatment outcome.

## 2. Materials and Methods

A systematic review of the literature was carried out in accordance with the PRISMA recommendations (PRISMA 2020 (Preferred Reporting Items for Systematic Reviews and Meta-Analyses; The PRISMA 2020 statement: an updated guideline for reporting systematic reviews [9]. The data were reported following the structure and content dictated by the 27 items included in the declaration and the review was carried out from December 2021 to June 2022. In addition, the review protocol has been registered in PROSPERO with the number CRD42022322926.

Those studies that compared different rotary file systems in primary teeth for the complete removal of the pulp, its cleaning and the subsequent obturation of the canals were included. The inclusion criteria were temporary teeth where root canal treatment was carried out with rotary systems. Subsequently, the results obtained from their use were assessed.

Randomized clinical trials (RCTs), longitudinal studies, cohort or case–control studies, both retrospective and prospective, were included. No restrictions were established regarding the year of publication or the language.

The objective was to answer the following research question: Which rotary instruments (C) offer better results (O) for root canal treatment (I) in patients with temporary dentition (P)?

In order to identify the most relevant studies, five different electronic databases were used: Pubmed, Embase, Scopus, Cochrane and Web of Science. In specific cases, the authors of the articles were contacted by email in order to request additional information. In addition, the references of the resulting studies were screened for potentially eligible studies that did not appear in the preliminary database search. This review was last updated as of June 2022.

The search strategy was designed considering previous studies in the field and their most cited descriptors. The keywords to identify the articles were: “Primary teeth” OR “pediatric dentistry” AND pulpectomy OR “root canal treatment” AND “rotary system”.

References identified by the search strategy were exported from each database to Mendeley’s reference management software (Elsevier, Amsterdam, The Netherlands) to check for duplicates. After ruling out duplicates, two reviewers (MD-CR and L-MM) independently assessed the titles and abstracts of all identified articles. In case of discrepancy between them, a third author (E-GM) was consulted. If the abstract did not provide enough information to make a decision, the reviewers read the full article. Finally, those who met the requirements were included in the study.

The data synthesis of the included studies was divided into variables for the study characteristics, methodology and results. For the identification of the characteristics of the studies, author and year were used. Regarding the methodology, the sample of treated teeth, the rotary systems used and its specific characteristics were evaluated. The outcome variables included the significant results found and the conclusions that were drawn from each study analyzed.

Studies included in this review were independently assessed for internal methodological risk of bias. The PEDro scale was used for experimental studies and RCTs.

## 3. Results

### 3.1. Study Selection and Flow Diagram: Study Results

**Figure 1:**
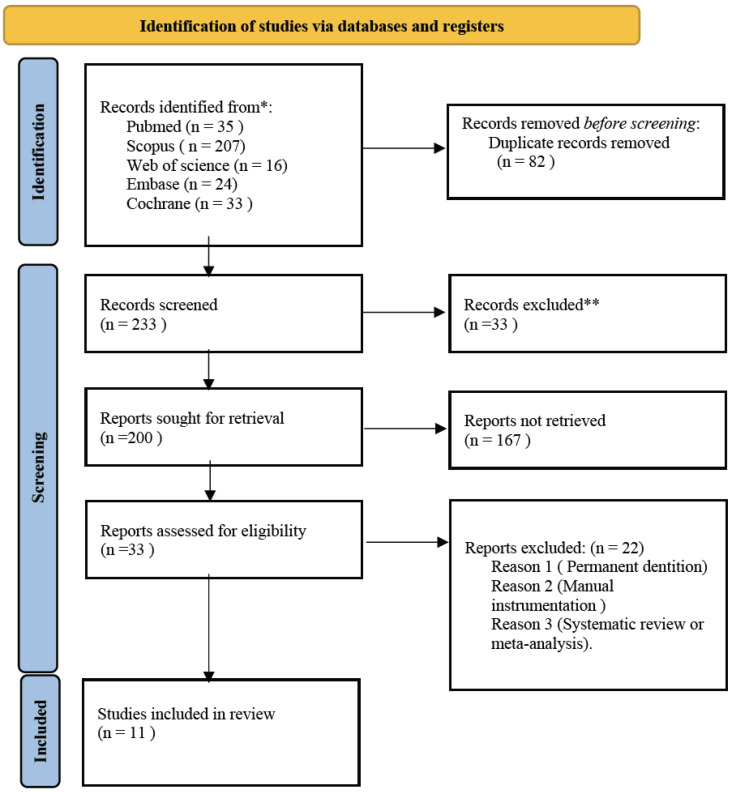
PRISMA flowchart of study selection process. * Consider, if feasible to do so, reporting the number of records identified from each database or register searched (rather than the total number across all databases/registers). ** If automation tools were used, indicate how many records were excluded by a human and how many were excluded by automation tools.

From the initial electronic search of the five databases, 315 articles were identified: 35 from Pubmed, 24 from Embase, 207 from Scopus, 33 from Cochrane and 16 from Web of Science. After eliminating the duplicate articles, a total of 233 remained. After reading the title and abstract, 200 articles were eliminated, leaving 33. Thanks to the reading of the full text, 22 were eliminated for not answering the research question or the inclusion criteria leaving a total of 11 articles for the systematic review. The PRISMA flowchart (Figure 1) provides an overview of the item selection process.

### 3.2. Results of Individual Studies, Meta-Analysis and Additional Analyses

Regarding the sample included in the studies obtained (Table 1 and Table 2), most of them analyzed an average of 30 teeth [10,11]. Highlighting Mohammadi, D [12], with a maximum of 80, and Moraes [13] with a minimum of 1. Regarding the variables analyzed, and considering the length of the files, the longest were those used by Gekelman [14], with a length 25 mm, and the shortest, 15 mm, used by Mohammadi [12]. It should be noted that the mean length used in most studies was 21 mm [15]. When assessing the diameter used, the mean was 25# [16], varying between a maximum of 50# [17] and a minimum of 20# [18]. Finally, when referring to file conicities, the most used are those of 0.06–0.07 [19], but others of 0.04 can be used [19], and even up to 0.20 [18].

Regarding the speed of the files, 300 rpm is usually recommended for performing pulpectomies. However, authors such as Mohammadi, D [12] have used higher speeds such as 1200 rpm.

Lastly, regarding the irrigant used, sodium hypochlorite is the most used at different concentrations: 2.5% [10], 5.25% [11] or 1% [11], although other authors also use saline solution to eliminate bacteria [13,18].

## 4. Discussion

Root canal treatment in primary teeth can be one of the biggest challenges in pediatric clinical practice. The anatomical particularities of the deciduous dentition, the difficulty of the behavior management during the instrumentation, the difficulty of using RPR and PRF therapies focused on endodontics in children, and eventually, the lack of understanding by parents of the need to preserve the primary teeth until its natural exfoliation add great difficulties to an already fairly complex procedure. Nevertheless, the premature loss of primary teeth will have functional and aesthetic consequences, which may negatively affect the general health of the pediatric patient [17,18].

Accurate determination of the pulp status will be key to the success of the treatment. It is necessary to consider that reversible pulpitis generally does not present symptoms or is less intense and shorter compared to irreversible pulpitis. In contrast, spontaneous, radiating pain that persists after removal of the stimulus tends to be indicative of irreversible pulpitis. Similarly, the absence of pain does not necessarily mean that the pulp is free of inflammation. Additionally, pediatric patients generally have a very low pain threshold compared to adults [20,21].

Necrosis will result from untreated irreversible pulpitis, traumatic injury, or any event that interrupts the blood supply to the pulp. This necrosis may be partial or total. When the necrosis is total, the treatment of choice will be a pulpectomy [22]. Different treatment options would be found, depending on the degree of pulp involvement, for immature permanent teeth where regenerative endodontics or vascular regeneration may be a choice prior to conventional endodontics [23].

Pulpectomy is considered the treatment of choice for deciduous dentition with irreversible involvement of the root pulp, as it allows the canal to be disinfected and completely sealed [24]. With success rates between 77–86%, pulpectomy is currently considered a highly reliable alternative to tooth extraction [19].

Over the years, the instruments used in endodontics have varied from manual to current rotary ones. However, the rotary files generally used by clinicians and most commonly described in the literature are designed specifically for endodontic treatment of permanent teeth and not for its use in the primary dentition. Furthermore, there is no specific protocol for rotary instrumentation in primary teeth [12].

Since the use of rotary instruments has proven to be faster, obtaining well-filled canals and uniform results with respect to manual instrumentation, the need arises to assess which rotary instruments are more effective in primary dentition, achieving a better three-dimensional preparation, cleaning and obturation of the canals [25]. Although there is the possibility of using alternative therapies such as light-activated disinfection for cleaning temporary root canals, which would be widely accepted by children and parents, this study will focus on the analysis of the different current rotary systems [26].

According to Kleier DJ [27], the ideal instrumentation technique for root canal preparation should be safe and performed effectively by previously trained clinicians. An efficient technique should be considered that creates apical stops, smoothens canal walls, shows good flow and an adequate conicity after preparation along with being able to carry out the treatment easily and quickly as well as creating a good root seal with minimum postoperative pain.

Due to the anatomy of temporary root canals (smaller size in any dimension compared to permanent ones but larger mesio-distal dimension, greater cervical constriction, very marked root grooves especially in molars, less thickness of the enamel and dentin) the manufacturing material of the files must have special characteristics. Of the files studied, the XP-endo shaper and finisher, as well as the Kedo SG blue or the Pro AF baby are both manufactured with an internal heat treatment which brings them greater flexibility. Being more flexible, they can adapt better to the root canal anatomy of the temporary teeth, significantly reducing the occurrence of complications as steps or perforations [21].

Similarly, the study of Govindaraju L [19] in 2018 explained that by reducing the time spent working in the dental chair, the cooperation of children for dental treatment increased. A year earlier, Jeevanandan G [28] observed that by using Kedo SG files, the clinical time was significantly reduced due to the decrease in the number of files during preparation. Likewise, and coinciding with the work of Yüksel B [10] in 2022, as shorter files were used (17 mm), a simpler and faster insertion into the canals was achieved, which facilitated their preparation.

The concept of single-instrument root canal preparation was introduced in 2008 and was quickly assimilated by endodontists around the world as it is a more efficient and faster technique compared to the use of multiple instruments. Authors such as Gekelman D [14] obtained good results for the study with RaCe files and the ProTaper system, both instrumented with a single file (size 25 for RaCe and S2 for Protaper). Similarly, Da Silva [29], compared the WaveOne Large (WO) and ProTaper F4 systems, for the preparation of temporary canals, finding better results in the preparation of the occlusal and middle third of WO compared to the PT group, although no significant differences were found.

Regarding the amount of dentin removed, for Azim AA [30] 2017, with the XP-endo Shaper system, more dentin was removed than with the Vortex Blue in the middle and coronal areas, but not in the apical area. In 2019, Tabbara A [21] reviewed the cleaning ability of BioRace systems by associating it with more virgin channel walls. However, Reciproc had the highest dentin removal results and the SAF system resulted in more damage of the root canal walls and less dentin removal. Finally, TRUShape showed intermediate results. None of the systems tested provided optimal modeling ability in oval shaped canals.

For Yüksel B [10], the rotary system that resulted in the highest rate of reduction in root dentin thickness was WaveOne Gold, followed by XP-endo Shaper and one shape. Regardless of the material used to seal the root canals, the quality of the filling will also depend on the type of instrumentation. In 2018, the study by Govindaraju L [19] did not observe differences in the quality of the obturation depending on the file used, which is in due to the high cleaning capacity of all rotary systems. On the contrary, Juliet S [16] in 2020 stated that the Kedo SG files, due to their larger coronal diameter, allow a better filling of the canals compared to other rotary systems and also affirmed that the RaCe system produced overfilling of the canals.

The intensity of the pain experienced by children clearly defines their behavior, and this will determine the final success of the treatment. According to Govindaraju L [19], a lower perception of root canal treatment has been observed in temporary dentition associated with the use of Kedo SG files, since their design adapts to the conicity of the deciduous dentition (0.40 mm in diameter at the tip and 16 mm in length), thus the subsequent files used during the preparation will end up being less damaging.

Closely linked to postoperative pain, extrusion of debris during root canal preparation is considered an important complication that should be kept in mind during the instrumentation of primary teeth. Such debris, in addition to increasing postoperative pain, are capable of producing a detrimental effect on the stem cells of the apical area [30]. In the study of Alnassar I [31] in 2021, it was concluded that manual instruments produced more debris than rotary instruments such as Protaper Next Waveone, without finding significant differences between both; [32] In the recent study by Yeung W-Y [33] 2022, the cross-sectional area of the instrument used was analyzed as one of the main parameters in determining the amount of extruded debris. In addition, it was also concluded that the volume of irrigant could condition said extrusion since a greater volume of irrigation will notably reduce the extrusion of the material.

For Souza RA [34] 2006, all the methods and instruments currently used produce a certain degree of extrusion of detritus. According to this author, they can be minimized by passing a small, pointed instrument that does not adhere to the apical area of the canal wall through the apical foramen.

Some authors, such as Juliet S [16] 2020, affirm that files such as the Kedo SG, due to the apical enlargement they generate, achieve a minimum extrusion of waste materials and, likewise, prevent the excessive preparation.

The Reciproc system, according to Labbaf H [35] 2017, resulted in a higher waste extrusion than other groups. This may be due to the S-shaped cross section and sharp blades, which provides it with a greater cutting capacity to remove more dentin wall material within the canal and thus produce more debris [36].

In 2019, Tabbara A [21] also compared the canal shaping ability of BioRace, Reciproc, SAF files and the TRUShape system, finding the same level of accumulated hard tissue debris.

According to Moraes M [17] 2021, the similarity for the resulting debris from WOG (wave one gold) and XPS (X-endo Shaper) was similar.

The above statements should be interpreted cautiously, as more reviews are needed regarding rotary instrumentation in primary dentition. The need for standardized studies, in vivo and ex vivo, where protocols for performing fast and efficient pulpectomies in primary dentition can be established is crucially important. Caries color, extent and symptomatology have the potential to be used as clinical diagnostic tools to determine pulp status in primary teeth. In the same way, a study on root canal filling materials for deciduous teeth would be interesting to be carried out in order to analyze the fractal dimension [37].

## 5. Conclusions

Rotary systems that are able to adapt to the root anatomy of primary teeth and allow rapid and simple instrumentation without producing excessive extrusion of debris at the root apex will be the ones, (for example Kedo SG files), that provide the best results to the pediatric dentist during the performance of root canal treatment in primary teeth.

## Figures and Tables

**Table 1:** List of the characteristics of the different current rotary systems for root canal treatment in temporary dentition.

Author, Year	n *	Limes	Length	Diameter	Taper	Rotation speed	Irrigant	Sealing material	Tracking time	Results
Yüksel B, 2022 [10]	30	One Shape system XP- endo^®^ Shaper WaveOne Gold system	17 mm 21 mm 21 mm	#25 #30 #25	0.06 0.04 0.07	400 rpm 800 rpm 800 rpm	2.5% sodium hypochlorite	N.A	6 months	One Shape lower danger value. >Microcracks in the middle of the root.
Vaishali D, 2021 [11]	30	Kedo SG Blue rotary files Pro AF Baby gold rotary files Pedo Flex rotary files	16 mm 17 mm 16 mm	#25, 30, 40 #20–40 #20–30	D1, E1, U1 0.06 0.04	300 rpm 300 rpm 350 rpm	10 mL of 1% sodium hypochlorite + saline + EDTA	zinc oxide eugenol (ZOE)	1 week	Kedo SG Blue > Optimal fillings and filled canals. <Gaps.
Mohammadi, D, 2021 [12]	80	Reciproc Protaper universal Hyflex CM Neolix	25 mm 21 mm 21 mm 15–21 mm	#25 #20–25 #25 #25	0.06 0.07/0.08 0.06/0.08 0.06/0.12	300 rpm. 500 rpm	Sodium hypochlorite and distilled water	N.A	2 days	Reciproc: ++Extrussion.
Moraes RDR, 2021 [13]	20	WaveOne^®^ GOLD XP-Endo^®^ Shaper XP-Endo^®^ Finisher XP Clean (XPC) System	21 mm 21 mm 21 mm 21 mm	#45 #30 #25 #25	0.05 0.04 - 0.02	Slowly 1000 rpm 1000 rpm 1000 rpm	0.9% saline solution	N.A	N.A	Acumulated debris, WOG y XPS +++. HF, remove + debris. XPC homogeneous. HF and XPC better instrumentation.
Gekelman D, 2009. [14]	20	GT rotary files ProTaper rotary files	25 mm 21 mm	S1,S2,F1	0.07/0.08	300 rpm	Tap water	N.A	N.A	No significative differences.
Boonchoo, K. 2020 [15]	37	WaveOne GoldTM	21 mm	#20	0.07	800 rpm	1% sodium hypochlorite	VitapexTM	1 y	Better filling of canals M.
Juliet, S. 2020 [16]	45	ProTaper Kedo-S RaCe	21 mm 16 mm	S1,S2,F1 #25–30 #25	0.07/0.08 D1, E1, U1 0.04	330 rpm 300 rpm	1 mL of 3% sodium hypochlorite + saline	Metapex	N.A	Kedo-S longer instrumentation time.
Moraes, R D R. 2019 [17]	1	Reciproc system	25 mm	#25 #40 #50	08 06 05	300 rpm.	Saline	N.A	N.A	R50 bigger risk of perforation. R40 Most valid option
Govindaraju L, 2018 [19]	30	ProTaper rotary file F1 Kedo-s rotary file	21 mm 16 mm	#20 #25–30	0.20/0.7 D1, E1, U1	330 rpm 300 rpm	Saline	Metapex	N.A	Kedo-S less postoperative pain. PT = KS preparation of the canal.
Marques da Silva, B. 2018 [20]	48	ProTaper rotary system f4 WaveOne Large	21 mm 25 mm	#40 #40	0.06 0.08	330 rpm	2.5% sodium hypochlorite 17%EDTA	N.A		No differences found between them.
Tabbara, A. 2019 [21]	20	XP-endo Shaper	21 mm	#30	0.04	800 rpm	Dakin’s solution 37 °C	Gutta-percha cone	N.A	The number of passes depends on the radicular anatomy. XP-endo Shaper obtains 30/0.04

n *: number of treated teeth.

**Table 2:** Analysis of the articles according to the PEdro scale—experimental studies and RCTs.

Items	Yüksel B, 2022 [10]	Vaishali D, 2021 [11]	Mohammadi, D, 2021 [12]	Moraes RDR, 2021 [13]	Gekelman D, 2009 [14]	Boonchoo, K. 2020 [15]	Juliet, S. 2020 [16]	Moraes, RDR. 2019 [17]	Govindaraju L, 2018 [19]	Marques da Silva, B. 2018 [20]	Tabbara, A. 2019 [21]
The selected criterio were specified	🟢 pg.3	🟢 pg.2	🟢 pg.2	🟢 pg.2	🟢v pg. 2	🟢 pg.2	🟢 pg.2	🟢**v** pg.2	🟢**v** pg.2	🟢**v** pg.2	🟢**v** pg.2
Sibjects were randomly assigned to group	🟢 pg.3	🟢 pg.2	🟢v pg.2	🟢v pg.5	🔴v pg.2	🟢 pg.2	🟢 pg.2	🔴**v** pg.2	🟢**v** pg.2	🔴**v** pg.2	🔴**v** pg.3
Concealed allocation of pactients	🔴 pg.3	🔴 pg.2	🔴 pg.2	🔴 pg.3	🔴v pg.2	🟢 pg.3	🟢 pg.2	🔴**v** pg.2	🟢**v** pg.2	🔴**v** pg.2	🔴**v** pg.2
Groups at baseline were similar in relation to the most important prognosis indicators	🟢 pg.3	🟢 pg.2	🟢 pg.4	🟢 pg.6	🟢 pg.3	🟢 pg.3	🟢 pg.3	🟢 pg.4	🟢 pg.3	🟢 pg.3	🟢 pg.3
All subjects were blinded	🔴 pg.3	🔴 pg.2	🔴 pg.2	🔴 pg.3	🔴v pg.2	🔴pg pg.3	🔴 pg.3	🔴**v** pg.2	🔴**v** pg.2	🔴**v** pg.2	🔴**v** pg.2
All clinicians were blinded	🔴 pg.3	🔴 pg.2	🔴 pg.2	🔴 pg.3	🔴v pg.2	🔴 pg.2	🔴 pg.3	🔴**v** pg.2	🔴**v** pg.2	🔴**v** pg.2	🔴**v** pg.2
All assessors were blinded	🔴 pg.3	🔴 pg.2	🔴 pg.2	🔴 pg.3	🔴v pg.2	🔴pg pg.2	🔴 pg.3	🔴**v** pg.2	🔴**v** pg.2	🔴**v** pg.2	🔴**v** pg.2
Means were obtained from more tan 85% of subjects	🟢 pg.4	🟢 pg.2	🟢 pg.4	🟢 pg.6	🟢 3pg.3	🟢 pg.3	🟢 pg.3	🟢 pg.4	🟢 3pg.3	🟢 3pg.3	🟢 pg.3
Results for all subjects were presented	🟢 pg.4	🟢 pg.2	🟢 pg.4	🟢 pg.6	🟢 pg.3	🟢 pg.3	🟢 pg.3	🟢 pg.4	🟢 pg.3	🟢 pg.3	🟢 pg.3
Statistical comparison results between groups were reported for at least one key outcome	🟢 pg.4	🔴 pg.2		🟢 pg.5	🔴 pg.5	🟢 pg.2	🟢 pg.3-4	🟢 pg.3	🟢 pg.3	🟢 pg.3	🟢 pg.3
The study provides one-off and variability measures for at least one key aoutcome	🟢 pg.5	🔴 pg.5		🟢 pg.7-8	🟢 pg.5	🟢 pg.3	🟢 pg.4	🟢 pg.4	🟢 pg.4	🟢 pg.3	🟢 pg.3
**Total**:	**7/11**	**5/11**		**7/11**	**6/11**	**8/11**	**8/11**	**6/11**	**6/11**	**4/11**	**6/11**
